# Free‐breathing self‐gated continuous‐IR spiral T1 mapping: Comparison of dual flip‐angle and Bloch‐Siegert B1‐corrected techniques

**DOI:** 10.1002/mrm.29269

**Published:** 2022-04-28

**Authors:** Ruixi Zhou, Junyu Wang, Daniel S. Weller, Yang Yang, John P. Mugler, Michael Salerno

**Affiliations:** ^1^ Department of Artificial Intelligence Beijing University of Posts and Telecommunications Beijing China; ^2^ Department of Biomedical Engineering University of Virginia Health System Charlottesville Virginia USA; ^3^ KLA Tencor, GPG/AI Charlottesville Virginia USA; ^4^ Biomedical Engineering and Imaging Institute and Department of Radiology Icahn School of Medicine at Mount Sinai New York New York USA; ^5^ Radiology & Medical Imaging, Biomedical Engineering University of Virginia Health System Charlottesville Virginia USA; ^6^ Department of Medicine, Cardiovascular Medicine and Department of Radiology, Cardiovascular Imaging Stanford University Palo Alto California USA; ^7^ Department of Medicine, Cardiology Division, Radiology and Medical Imaging, and Biomedical Imaging University of Virginia Health System Charlottesville Virginia USA

**Keywords:** B1 mapping, cardiac MRI, dictionary learning, free‐breathing, motion‐correction, self‐gating, spiral trajectory, T1 mapping

## Abstract

**Purpose:**

To develop a B1‐corrrected single flip‐angle continuous acquisition strategy with free‐breathing and cardiac self‐gating for spiral T1 mapping, and compare it to a previous dual flip‐angle technique.

**Methods:**

Data were continuously acquired using a spiral‐out trajectory, rotated by the golden angle in time. During the first 2 s, off‐resonance Fermi RF pulses were applied to generate a Bloch‐Siegert shift B1 map, and the subsequent data were acquired with an inversion RF pulse applied every 4 s to create a T1* map. The final T1 map was generated from the B1 and the T1* maps by using a look‐up table that accounted for slice profile effects, yielding more accurate T1 values. T1 values were compared to those from inversion recovery (IR) spin echo (phantom only), MOLLI, SAturation‐recovery single‐SHot Acquisition (SASHA), and previously proposed dual flip‐angle results. This strategy was evaluated in a phantom and 25 human subjects.

**Results:**

The proposed technique showed good agreement with IR spin‐echo results in the phantom experiment. For in‐vivo studies, the proposed technique and the previously proposed dual flip‐angle method were more similar to SASHA results than to MOLLI results.

**Conclusions:**

B1‐corrected single flip‐angle T1 mapping successfully acquired B1 and T1 maps in a free‐breathing, continuous‐IR spiral acquisition, providing a method with improved accuracy to measure T1 using a continuous Look‐Locker acquisition, as compared to the previously proposed dual excitation flip‐angle technique.

## 1 INTRODUCTION

Heart failure (HF) is a major and growing public health problem worldwide. Determination of the underlying cause of a patient's HF syndrome has important diagnostic, therapeutic, and prognostic implications. Cardiac magnetic resonance (CMR) imaging is increasingly used to assess the etiology of HF and to assess for specific cardiomyopathies. Among the different techniques, cardiac *T*_1_ mapping enables both qualitative and quantitative assessment of myocardium, and has demonstrated the ability to evaluate both focal and diffuse myocardial processes in cardiomyopathy.^1^, ^2^, ^3^

In current clinical practice, *T*_1_ maps are typically acquired before and after contrast using a Modified Look‐Locker‐Inversion recovery (MOLLI) technique.^4^ It acquires single‐shot images intermittently in diastole during three‐five heartbeats after each of multiple inversion recovery (IR) pulses with breath‐holding and electrocardiograph (ECG)‐gating. Alternatively, the SAturation‐recovery single‐SHot Acquisition (SASHA) sequence^5^ acquires breath‐held single‐shot saturation recovery prepared images in each of multiple heartbeats. When both pre‐ and post‐contrast *T*_1_ maps are acquired, and hematocrit is measured, extracellular volume (ECV) fraction can be calculated.

Recently, multiple studies^6^, ^7^, ^8^ have proposed acquiring *T*_1_ maps with free‐breathing and/or without ECG‐gating. However, to use a continuous Look‐Locker acquisition, where cardiac self‐gating can be achieved, both $B_{1}^{+}$ and slice profile effects^9^, ^10^ need to be considered to accurately quantify *T*_1_. Previously, we proposed a technique to obtain *T*_1_ and flip‐angle scale maps from a single free‐breathing self‐gated continuous IR‐based acquisition using dual excitation flip angles^11^ (2FAs CAT‐SPARCS). To provide an alternative approach with improved *T*_1_ accuracy, in this study we proposed a Bloch‐Siegert (BS)^12^ shift $B_{1}^{+}$‐corrected single flip angle acquisition under free‐breathing and cardiac self‐gating (1FA + $B_{1}^{+}$), where a separate $B_{1}^{+}$ map was acquired during free breathing and used in calculating the final *T*_1_ map.

Most studies^6^, ^13^ have compared the in‐vivo *T*_1_ results with those from MOLLI, which is known to underestimate *T*_1_ values in myocardium. Similarly, ECV results generated from MOLLI *T*_1_ maps also suffer from a bias with an overestimation of ECV by MOLLI as compared to SASHA.^14^ In this study, we compared the proposed 1FA + $B_{1}^{+}$ CAT‐SPARCS method to the 2FAs CAT‐SPARCS technique, as well as to IR spin echo (phantom only), MOLLI, and SASHA in phantom and in‐vivo experiments.

## 2 METHODS

### 2.1 Image acquisition

**FIGURE 1:**
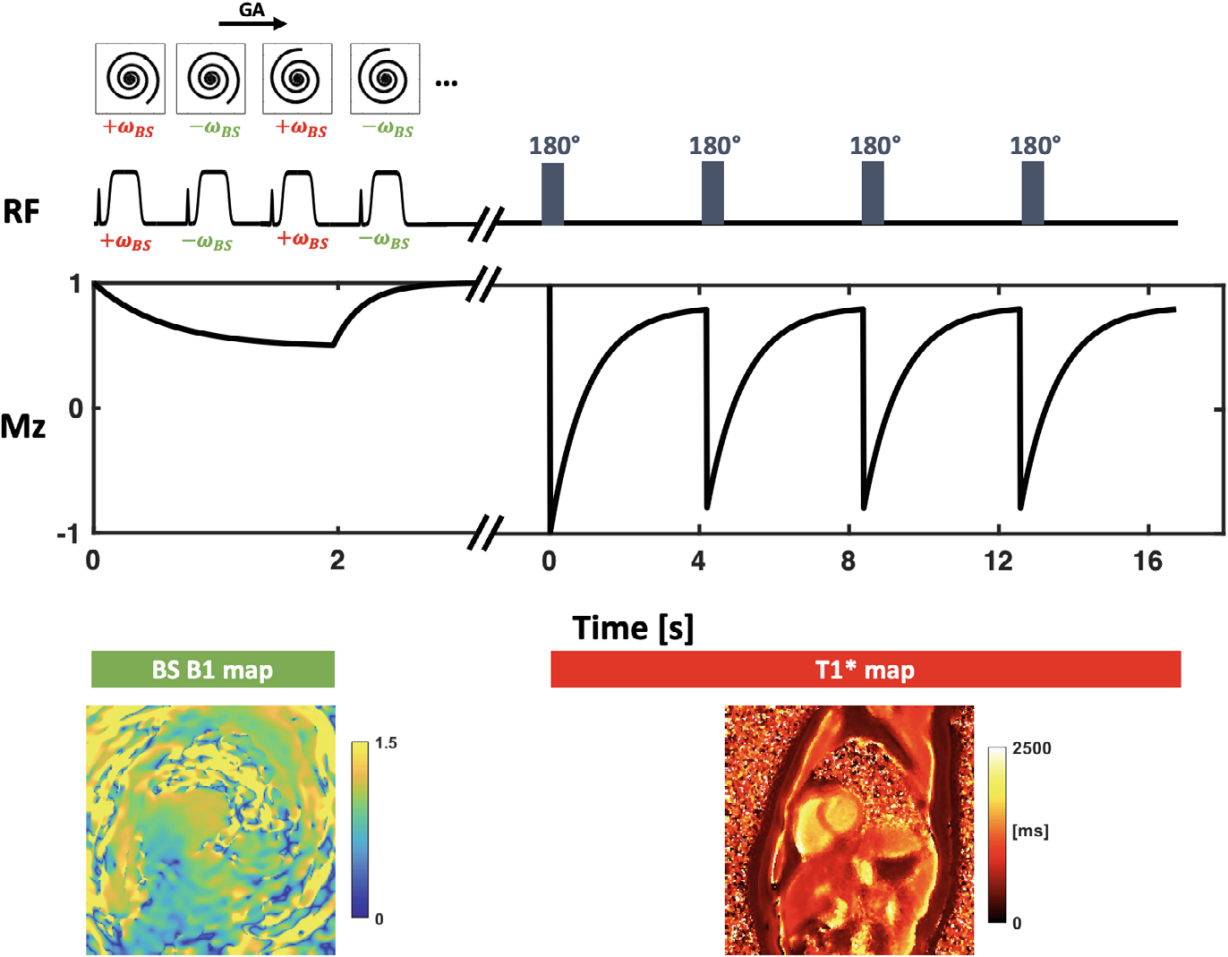
General schematic of the acquisition. All data are acquired using a spiral‐out *k*‐space trajectory rotated in time by the golden angle (GA). During the first 2 s, data are acquired in combined with off‐resonance Fermi pulses, which applied at frequencies +ω_*BS*_ and −ω_*BS*_. This was used to reconstruct the BS B1^+^ map. Using another sequence, remaining data are acquired with inversion pulses applied at 4 s intervals, and used to reconstruct the T1* map

**FIGURE 2:**
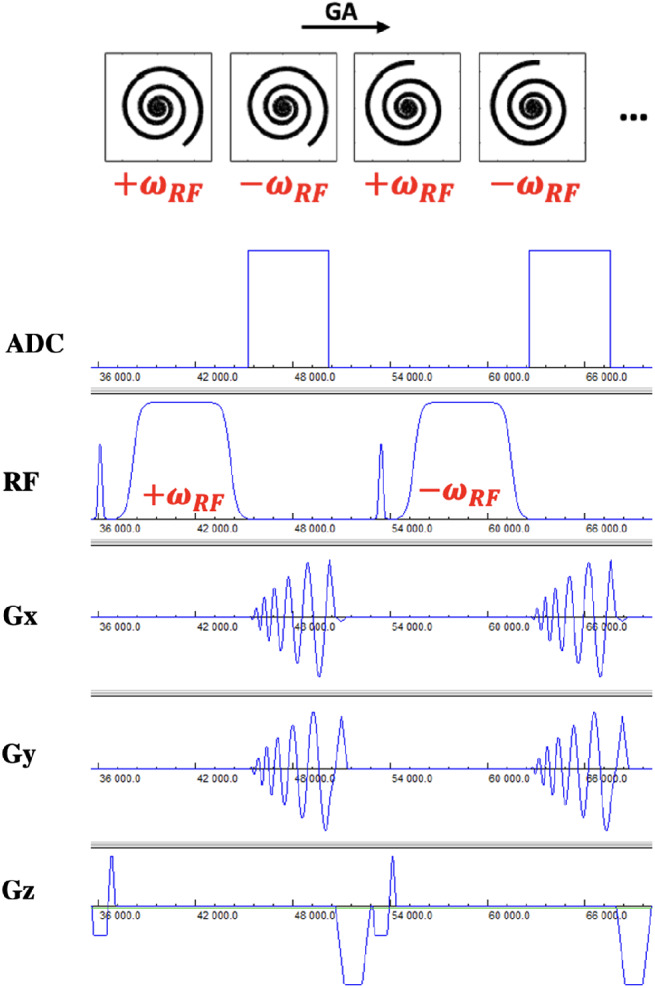
Sequence diagram depicting the spiral BS B1^+^ mapping sequence. Images with positive and negative phase shifts were acquired in an interleaved fashion

As shown in Figure 1, for BS shift $B_{1}^{+}$ mapping,^12^ data were acquired continuously with the golden‐angle rotated spiral‐out trajectories for 2 s, and the TR was set to the minimal TR that met specific absorption rate (SAR) limitations. For each repetition during this period, an off‐resonance Fermi pulse was applied between the slice rewinder and spiral readout‐gradients, as shown in Figure 2. In order to cancel the background and undesired phases, and also to minimize the impact of motion between images obtained with different BS shifts, positive (+ω_*BS*_) and negative (−ω_*BS*_) off‐resonance Fermi pulses were interleaved among spiral trajectories. Currently, a single BS $B_{1}^{+}$ map is acquired only once at pre‐contrast for each subject. In a separate sequence for $T_{1}^{*}$ mapping, following an IR RF pulse, golden‐angle spiral‐out trajectories were acquired continuously over 4 s using a spoiled‐GRE pulse sequence. This pattern was repeated four times. In the 2FAs approach, the IR‐acquisition portion of the pulse sequence was repeated with a second flip angle, as described previously.^15^

### 2.2 Image reconstruction

For the BS $B_{1}^{+}$ map, images were reconstructed using non‐uniform fast Fourier transform (NUFFT)^16^ and Walsh coil combination^17^ without cardiac‐gating. The BS phase shift was generated by irradiating with an off‐resonance RF pulse following excitation. The phase shift can be expressed as^12^:

(1) $$\varphi_{\mathrm{BS}}=B_{\left(1,\text{peak}\right)}^{2}\int_{0}^{T_{\text{pulse}}}\frac{{\left(\gamma B_{1,\mathrm{norm}}\left(t\right)\right)}^{2}}{2\omega_{\mathrm{BS}}\left(t\right)}\mathrm{dt}=B_{1,\text{peak}}^{2}\cdot K_{\mathrm{BS}},$$

where $B_{1,\text{peak}}$ is the maximum $B_{1}$ magnitude of the RF waveform, and the factor $K_{\mathrm{BS}}$ is computed from the normalized BS pulse shape *B*_1,norm_ (t) with duration *T*_pulse_, the off‐resonance frequency $\omega_{\mathrm{BS}}\left(t\right)$ and the gyromagnetic ratio γ.

**FIGURE 3:**
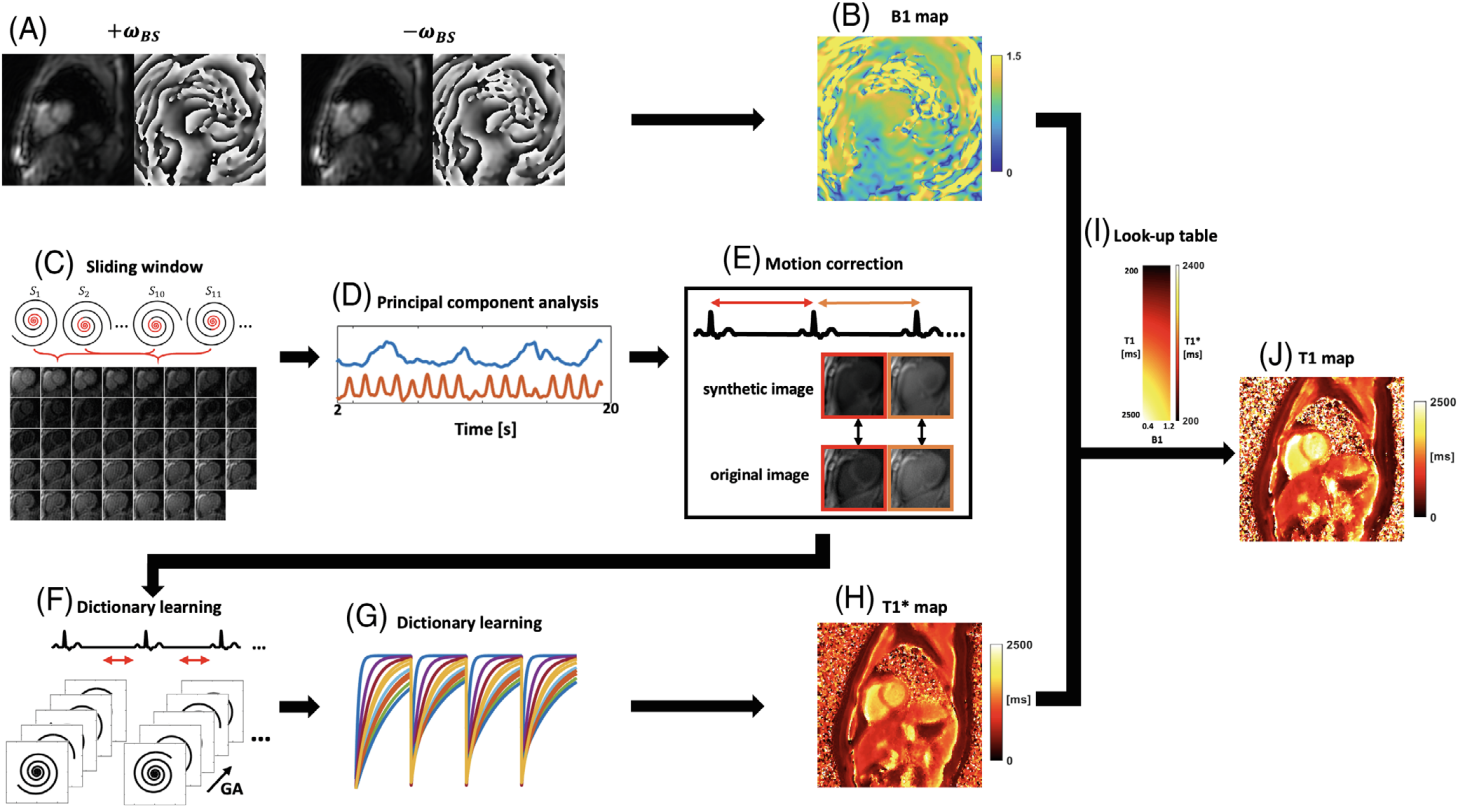
Image processing strategy. A, BS magnitude and phase images are shown. The phase images (A) corresponding to positive and negative frequency shifts were extracted to calculate the BS shift B1^+^ map (B). Using the data collected following inversion pulses, low‐resolution image navigators (C) were generated using a sliding window approach, from which self‐gated cardiac triggers (D) were extracted using principal component analysis. E, Then, respiratory motion was corrected by rigid registration on each heartbeat. F, Next, data in the diastolic acquisition window for each R‐R interval were combined. A dictionary (G) was generated based on the acquisition parameters, a range of T1 and B1^+^ values, then dictionary learning reconstruction was performed to create the T1* map (H). T1 map (J) was generated using the B1^+^ map and T1* map through the look‐up table matching (I)

The positive (*I*_+_) and negative (*I*_−_) off‐resonance images are proportional to the magnitude of the magnetization M, the background phase $\varphi_{0}$, and the BS phase shift $\varphi_{\mathrm{BS}}$. Representative magnitude and phase images are shown in (Figure 3A). The two images can be formulated as^18^:

(2) $$I_{+}\propto |M|e^{j\left(\varphi_{0}+\varphi_{\mathrm{BS}}\right)},$$

(3) $$I_{-}\propto |M|e^{j\left(\phi_{0}-\phi_{\mathrm{BS}}\right)},$$

The $B_{1}^{+}$ map (Figure 3B) is given as the peak value of the BS pulse *B*_1,peak_:

(4) $$B_{1,\text{peak}}=\sqrt{\frac{\arg\left(I_{+}/I_{-}\right)}{2K_{\mathrm{BS}}}}=\sqrt{\frac{\varphi_{\mathrm{BS}}}{K_{\mathrm{BS}}}},$$

As described previously,^15^ the $T_{1}^{*}$ map self‐gating cardiac triggers were extracted from a sliding‐window heart image navigator (Figure 3C,D). Respiratory motion correction was performed by rigidly registering the original image with its corresponding synthetic image that is generated from principal component analysis (PCA), as they shared similar image contrast (Figure 3E). Then, every five spirals were combined to create a set of four images each with a 42 ms temporal resolution during the diastolic acquisition window for each R‐R interval (Figure 3F). Dictionary learning reconstruction was performed to remove residual aliasing (Figure 3G, Supporting Information Figure S1, which is available online). When the heartrate is 60 bpm, the number of images used to fit the three‐parameter model getting $T_{1}^{*}$ map will be 64. Furthermore, to align the heart location of the acquired $B_{1}^{+}$ map with that for the generated $T_{1}^{*}$ map, a rigid registration was performed on the magnitude images from BS $B_{1}^{+}$ map and $T_{1}^{*}$ map around the heart region of interest (ROI).

To account for slice profile and $B_{1}^{+}$ effects, the dictionaries were generated by simulating 200 isochromats across the actual slice profile (windowed sinc pulse with time‐bandwidth [TBW] = 5.4). Using the proposed acquisition parameters assuming ideal IR pulse with the exact acquisition length, the isochromats were simulated across the slice profile for a range of $T_{1}$ values from 200 ms to 2500 ms and $B_{1}^{+}$ scales from 0.4 to 1.2 to create a look‐up table including slice profile effects. Finally, a *T*_1_ map was generated by matching the $B_{1}^{+}$ and $T_{1}^{*}$ maps using the look‐up table (Figure 3I). A detailed relationship between $B_{1}^{+}$, $T_{1}^{*}$, and *T*_1_ of simulated look‐up table can be visualized in Supporting Information Figure S2.

### 2.3 Phantom study

Imaging experiments were performed at 3T (MAGNETOM Prisma; Siemens Healthineers) using a T1MES phantom.^19^ *T*_1_ maps were generated using the previous 2FAs technique and the proposed 1FA + $B_{1}^{+}$ strategy, and compared to those from IR‐SE, MOLLI,^4^ and SASHA.^5^ The sequence parameters for the 2FAs technique included: TR/TE = 8.35/1.45 ms, RF pulse TBW = 5.4, FOV = 340 × 340 mm^2^, spatial resolution = 1.5 × 1.5 × 8 mm^3^, flip angle = 3° for the first four IR pulses and flip angle = 15° for the last four IR pulses. A dual‐density spiral‐out trajectory was used with a Fermi‐function transition region, where a *k*‐space density of 0.2 times Nyquist sampling was used for the first 20% of the trajectory and an ending density was 0.026 times Nyquist.^20^ For the 1FA + $B_{1}^{+}$ technique, BS $B_{1}^{+}$ maps were acquired with the following parameters: Fermi RF‐pulse duration = 8 ms, off‐resonance shift = ±4 kHz, *K*_BS_ = 79.73 $\mathrm{rad}/G^{2}$, *B*_1,peak_ = 0.0544 G, FOV = 340 × 340 mm^2^, spatial resolution = 1.5 × 1.5 × 8 mm^3^, TR/TE = 40.2/9.14 ms, flip angle = 15°, and reconstructed with spatial resolution = 10 × 10 × 8 mm^3^. To directly acquire $B_{1}^{+}$ maps with 10 × 10 × 8 mm^3^ resolution, the starting and ending densities of the spirals were 1.0‐ and 0.6‐times Nyquist sampling, respectively. In order to compare the $B_{1}^{+}$ mapping with a conventional technique, a fully‐sampled Cartesian BS $B_{1}^{+}$ map was separately acquired, and the parameters included: Fermi RF‐pulse duration = 8 ms, off‐resonance shift = ±4 kHz, *K*_BS_ = 79.73 rad/G^2^, *B*_1,peak_ = 0.1088 G, FOV = 340 × 340 mm^2^, resolution = 1.5 × 1.5 × 8 mm^3^, TR/TE = 107.5/30 ms, flip angle = 15°. The $T_{1}^{*}$ mapping portion of the pulse sequence was identical to the first half of the 2FAs technique.

To obtain reference *T*_1_ values of the phantom for validation, a 2D IR‐SE sequence was performed as the gold‐standard with the following parameters: FOV = 190 × 190 mm^2^, spatial resolution = 1.5 × 1.5 × 8 mm^3^, TR/TE = 10 000/10 ms, and 15 TI values = 50, 100, 150, 200, 250, 300, 400, 500, 750, 1000, 1200, 1500, 1700, 2000, and 3000 ms. Reference *T*_1_ values were determined by a three‐parameter non‐linear least‐squares fitting algorithm. In addition, a clinically used 5(3)3 MOLLI sequence with balanced SSFP (bSSFP) readouts was performed with the following imaging parameters: FOV = 340 × 340 mm^2^, resolution = 1.5 × 1.5 × 8 mm^3^, TR/TE = 2.61/1.08 ms, flip angle = 35°, parallel imaging (GRAPPA) acceleration with *R* = 2, and 6/8 partial Fourier. SASHA $T_{1}$ mapping was also performed, and parameters included: FOV = 340 × 340 mm^2^, resolution = 1.5 × 1.5 × 8 mm^3^, TR/TE = 2.9/1.24 ms, flip angle = 70°, parallel imaging (GRAPPA) acceleration with *R* = 2, and 7/8 partial Fourier. Images were acquired during an end‐diastolic window of around 167 ms. The ECG signal was simulated on the scanner at a heart rate of 60 beats/min.

### 2.4 In‐vivo study

The in‐vivo experiments were performed at 3T (MAGNETOM Prisma or Skyra; Siemens Healthineers) in 10 healthy volunteers and 15 patients undergoing clinically ordered CMR studies (11 males, 14 females; age: 50 ± 16). The patient group includes three subjects with coronary artery disease, one with pulmonary atresia, two with diabetes, and nine with cardiomyopathy. Three healthy volunteers underwent only pre‐contrast scans, while the rest of the healthy volunteers and patients received a gadolinium (Gd)–based contrast (gadoteric acid—gadoterate meglumine, Clariscan GE Healthcare) during the scan to perform both pre‐ and post‐contrast acquisitions. The seven healthy volunteers who received contrast agent were scanned twice (2FAs and 1FA + $B_{1}^{+}$) for repeatability testing. Short‐axis base and middle slices were acquired. All subjects gave written informed consent or had waiver of written informed consent with consent obtained by a phone call, and imaging studies were performed under institutional review board (IRB) approved protocols. *T*_1_ maps were obtained using the proposed strategy (1FA + $B_{1}^{+}$) and the previous technique (2FAs), and compared with those from MOLLI^4^ and SASHA.^5^ In order to speed up the acquisition, we only acquired the 2FAs technique and a separate BS $B_{1}^{+}$ map in the in‐vivo studies. The 3‐degree $T_{1}^{*}$ map is the same in the 1FA +$\;B_{1}^{+}$ and 2FAs approaches to avoid another impact factor when comparing the two. Since the injection of contrast will not affect the $B_{1}^{+}$ mapping, BS $B_{1}^{+}$ map was only acquired once at pre‐contrast and shared with post‐contrast acquisitions. Sequence parameters were the same as those described for the phantom experiments, except that the TR for BS $B_{1}^{+}$ portion ranged from 23.8 ms to 43.1 ms among subjects due to SAR limits.

### 2.5 Image analysis

Image reconstruction, processing and statistical analysis were performed using MATLAB (The Mathworks Inc.).

*T*_1_ values were compared by drawing ROIs in different tubes for the analysis of the phantom data. For human subjects, an ROI was drawn to include the whole myocardium on a basal short‐axis slice, and a second ROI was drawn in the blood pool with effort to avoid including papillary muscles. For phantom results, the mean *T*_1_ values were compared among techniques, the mean values from 2FAs, 1FA + $B_{1}^{+}$, MOLLI, and SASHA were correlated against those from IR‐SE, and Bland–Altman plots of *T*_1_ values were made to compare the 1FA + $B_{1}^{+}$, 2FAs, MOLLI and SASHA techniques with the IR‐SE method. The in‐vivo *T*_1_ values were plotted in box and whisker plots. To compare the *T*_1_ map image quality, we asked an experienced cardiologist to blindly grade the *T*_1_ maps among the four techniques on a 5‐point scale ranging from 1 (poor and not usable) to 5 (clinically excellent). A score of 3 is clinically acceptable, but with some artifacts. Two‐way analysis of variance (ANOVA) was used to perform the statistical test when compared the four techniques, considering the variance across the subjects.

## 3 RESULTS

**FIGURE 4:**
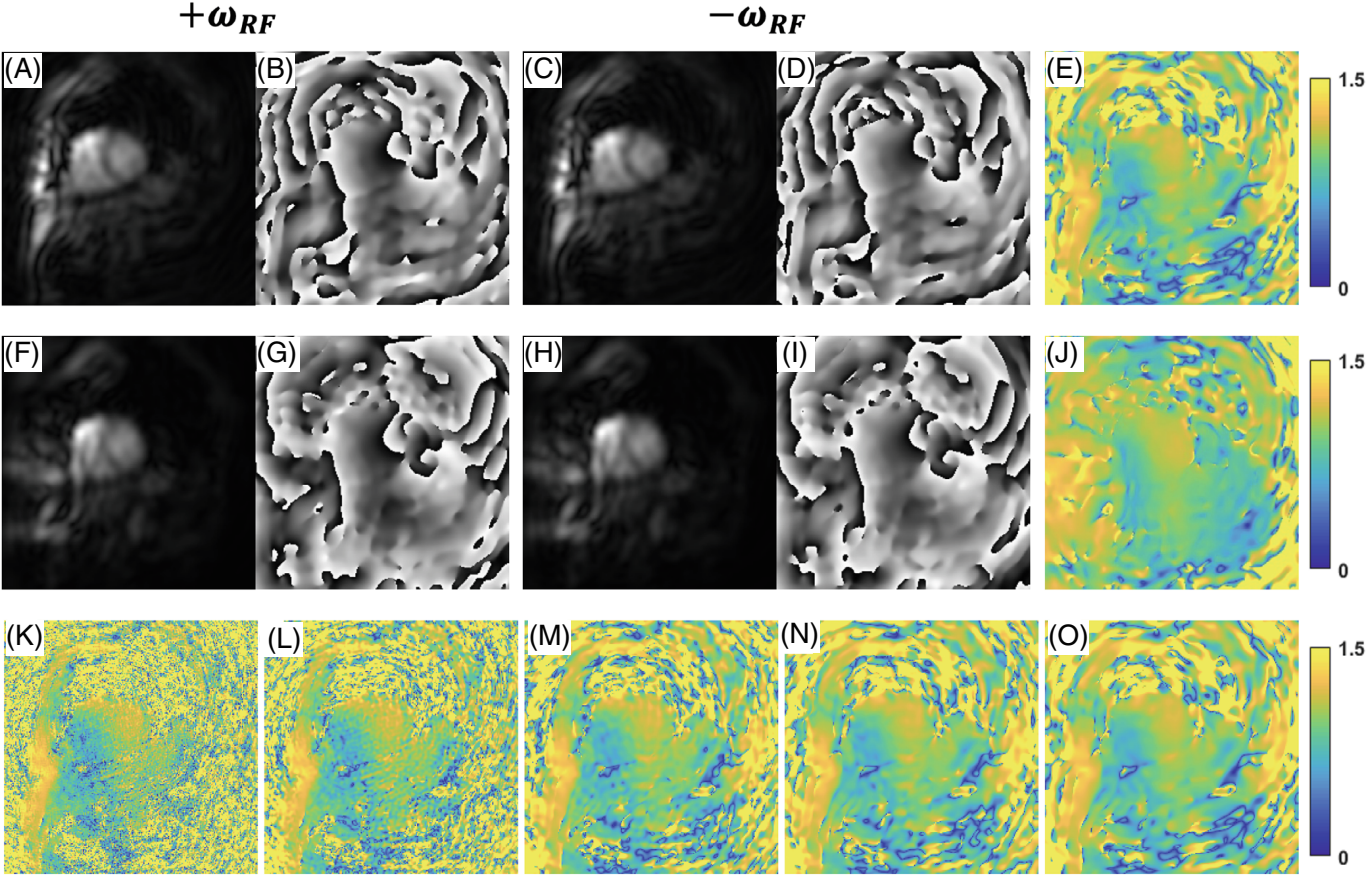
B1^+^ maps with different in‐plane spatial resolutions. A‐E, Acquired with 1.5 mm in‐plane spatial resolution and reconstructed with 10 mm resolution. F‐J, Acquired and reconstructed with 10 mm in‐plane spatial resolution. A,B,F,G, Positive magnitude and phase images corresponding to the positive frequency shift. C,D,H,I, Magnitude and phase images corresponding to the negative frequency shift. E,J, The resulting B1^+^ maps. K‐O, The acquired 1.5 mm in‐plane resolution and reconstructed at 1.5, 3, 6, 8, 10 mm

Figure 4 shows the $B_{1}^{+}$ maps acquired at different spatial resolutions. The top row images (A‐E) were acquired at 1.5 × 1.5 mm^2^ in‐plane spatial resolution and reconstructed at 10 × 10 mm^2^, while the middle row images (Figure 4,F‐J) were acquired and reconstructed at 10 × 10 mm^2^ in‐plane spatial resolution. The bottom row images (Figure 4K‐O) are the $B_{1}^{+}$ maps acquired at 1.5 × 1.5 mm^2^ resolution and reconstructed at 1.5 × 1.5 (K), 3 × 3 (L), 6 × 6 (M), 8 × 8 (N), 10 × 10 (O) mm^2^. Acquiring BS $B_{1}^{+}$ maps at a relatively high in‐plane spatial resolution of 1.5 × 1.5 mm^2^ provides the opportunity to reconstruct the final maps at multiple lower spatial resolutions that allowed us to determine an appropriate spatial resolution for $B_{1}^{+}$ mapping. Once an appropriate spatial resolution is chosen, acquiring at a lower resolution increases the SNR as seen in the figure. The mean SNR of the magnitude images for BS $B_{1}^{+}$ map acquisition between 1.5 × 1.5 mm^2^ and 10 × 10 mm^2^ resolution are 88.9 and 478.1 across five subjects.

**FIGURE 5:**
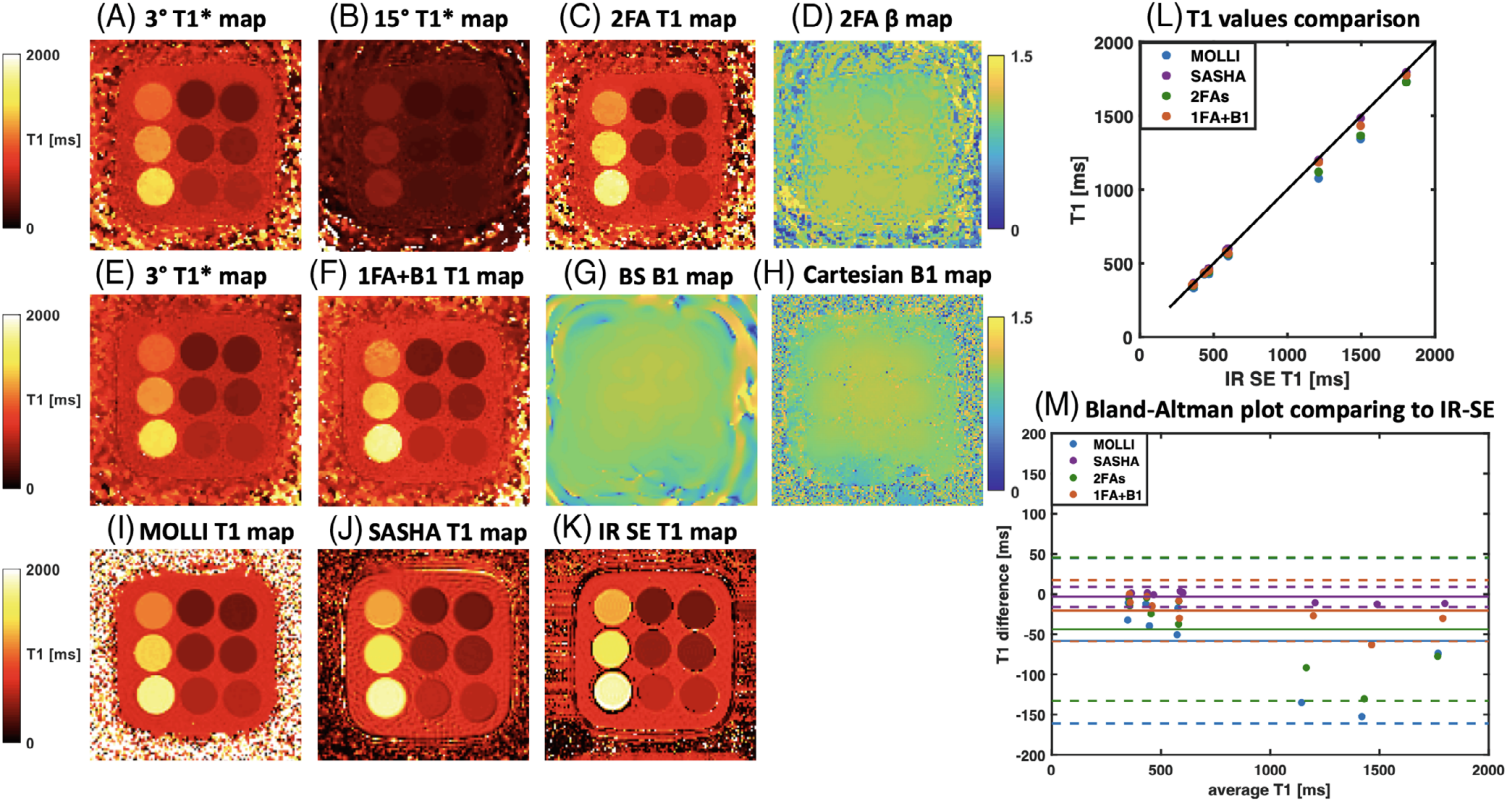
Phantom results. The 3° (A) and 15° (B) T1* maps from the 2FAs method were used to generate the T1 map (C) and flip angle scale‐factor (β) map (D). The 3° T1* map (E) and BS shift B1^+^ map (G) from the 1FA + B1^+^ method were used to generate T1 map (F). H, A separately acquired conventional Cartesian BS B1^+^ map as a comparison. The proposed T1 maps from the 2FAs and 1FA + B1^+^ methods were compared to those from MOLLI (I), SASHA(J), and IR‐SE (K). L, The T1 values from ROIs in each of the nine tubes for the 2FAs, 1FA + B1^+^, MOLLI, and SASHA techniques were compared to IR‐SE. M, A Bland–Altman plot comparing the 2FAs, 1FA + B1^+^, MOLLI, and SASHA techniques with IR‐SE

Figure 5 summarizes the phantom results from the 2FAs (Figure 5A–D) and 1FA + $B_{1}^{+}$ (Figure 5E–G) techniques. For 1FA + $B_{1}^{+}$ technique, $B_{1}^{+}$ map (Figure 5G) was compared to a conventional Cartesian BS $B_{1}^{+}$ map (Figure 5H). The 2FAs $T_{1}$ map (Figure 5C) and 1FA+$B_{1}^{+}$ *T*_1_ map (Figure 5F) were compared to those from MOLLI, SASHA, and IR‐SE (Figure 5I–K). The phantom $T_{1}$ values of nine tubes from 2FAs and 1FA + $B_{1}^{+}$ were in close agreement with the IR‐SE results (Figure 5L). The 2FAs, 1FA + $B_{1}^{+}$, and SASHA methods showed better agreement with IR‐SE than MOLLI did. For the 1FA + $B_{1}^{+}$ technique, the highest bias for the long $T_{1}$ tube was less than 5% (Figure 5M). The 95% limits of agreement are shown in separate Bland–Altman plots in Supporting Information Figure S3. Taking the slice profile effect into consideration further improves the $T_{1}$ accuracy as compared to the maps that did not include slice profile correction (Supporting Information Figure S4). In terms of $B_{1}^{+}$ quantification, although there are some minor differences between the BS $B_{1}^{+}$ map and the beta map generated from 2FAs technique, the normalized RMS error (NRMSE) between proposed β map and BS $B_{1}^{+}$ map across nine tube ROIs is 0.08, while the NRMSE between the BS $B_{1}^{+}$ map and Cartesian $B_{1}^{+}$ map across nine tube ROIs is 0.03.

**FIGURE 6:**
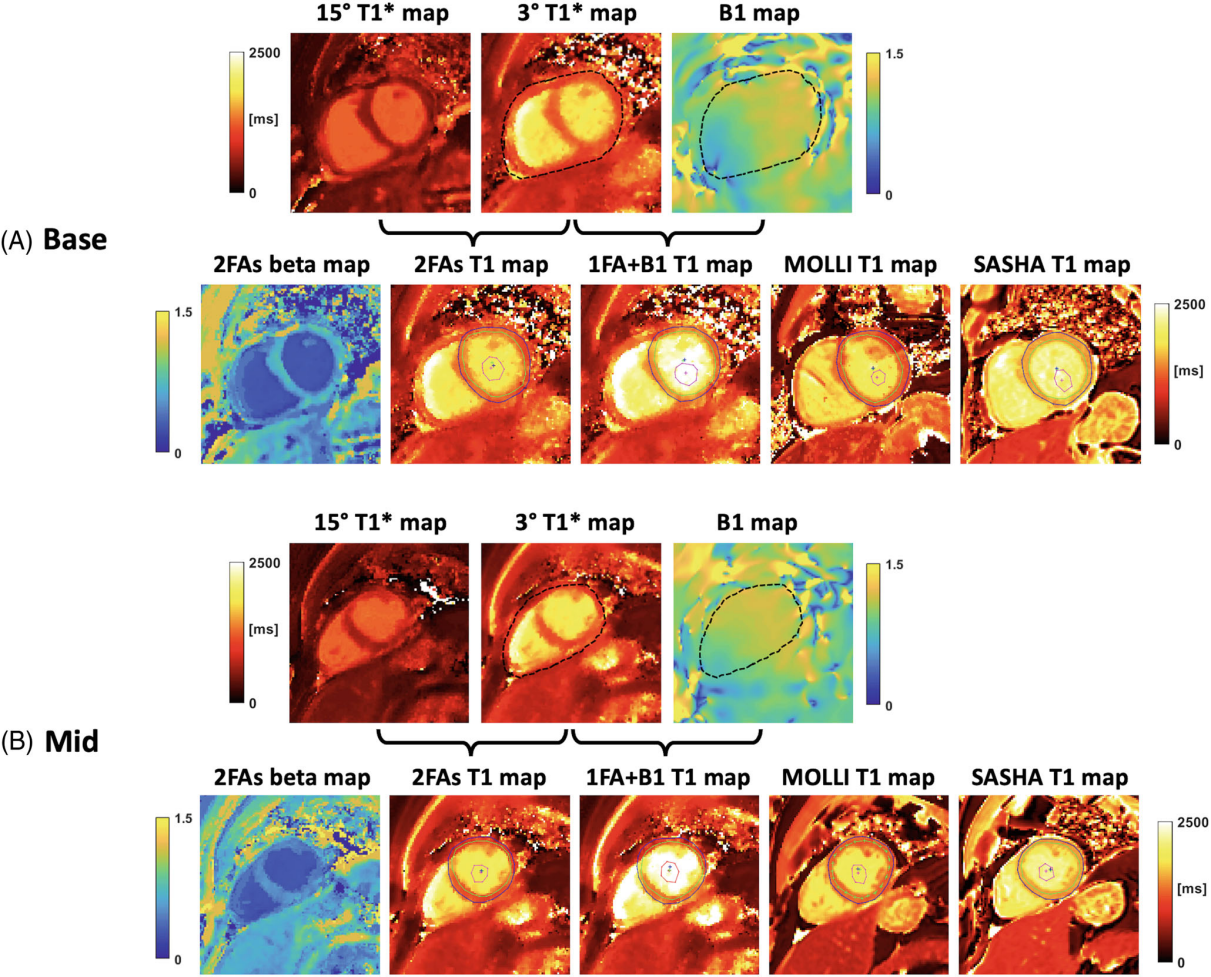
Healthy volunteer example. Short‐axis basal (A) and middle (B) slices from a healthy volunteer are shown. As indicated by the brackets, 3° and 15° T1* maps are used to generate the 2FAs T1 map, while 3° T1* and BS B1^+^ maps are used to generate the 1FA + B1^+^ T1 map. MOLLI and SASHA T1 maps are shown for comparison. The black dashed contours in T1* and B1^+^ map indicate the epicardial borders. The colored curves in T1 maps indicate the drawn ROIs. The mean ± SD of myocardium T1 values for 2FAs, 1FA + B1, MOLLI, and SASHA are 1226 ± 105.2 ms, 1378 ± 137.1 ms, 1170.7 ± 142.6 ms, and 1513.2 ± 62.72 ms

**FIGURE 7:**
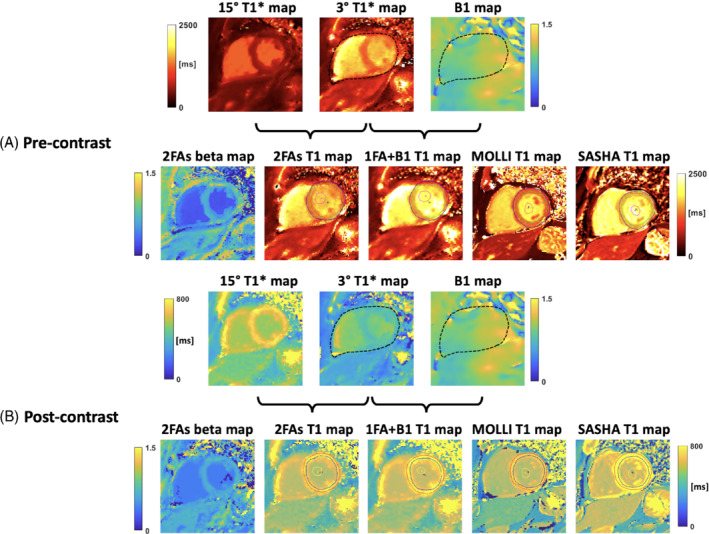
Patient example. Short‐axis basal slices from pre‐contrast (A) and post‐contrast (B) acquisition for a patient with coronary artery disease are shown. As indicated by the brackets, 3° and 15° T1* maps are used to generate the 2FAs T1 map, while 3° T1* and BS B1^+^ maps are used to generate the 1FA + B1^+^ T1 map. MOLLI and SASHA T1 maps are shown for comparison. The black dashed contours in T1* and B1^+^ map indicate the epicardial borders. The colored curves in T1 maps indicate the drawn ROIs. The mean ± SD of pre‐contrast myocardium T1 values for 2FAs, 1FA + B1, MOLLI, and SASHA are 1206 ± 222.7 ms, 1349.4 ± 253.3 ms, 1226.2 ± 167 ms, and 1558.7 ± 124.7 ms. The mean ± SD of post‐contrast myocardium T1 values for 2FAs, 1FA + B1, MOLLI, and SASHA are 745.7 ± 39.8 ms, 789 ± 45.5 ms, 564.8 ± 30.6 ms, and 920.8 ± 46.6 ms

Figure 6 shows results of a healthy volunteer at short‐axis basal (Figure 6A) and mid‐ventricular (Figure 6B) slices. The 2FAs and 1FA + $B_{1}^{+}$ $T_{1}$ maps were compared to those from MOLLI and SASHA. Figure 7 shows results of a patient with coronary artery disease at a short‐axis basal slice pre‐ (Figure 7A) and post‐contrast (Figure 7B). The 2FAs and 1FA + $B_{1}^{+}$ $T_{1}$ maps were compared to those from MOLLI and SASHA. By comparison, myocardium $T_{1}$ values from 1FA + $B_{1}^{+}$ are similar to SASHA myocardium $T_{1}$ results, but the proposed technique suffers residual artifacts. Artifacts in the inferior region at 3T might result from the B0 inhomogeneity. The comparison of the myocardium $B_{1}^{+}$ values from $B_{1}^{+}$ map and beta map can be seen in Supporting Information Figure S5. Of note, the beta values were lower in the blood pool for the 2FAs map (mean: 1.06 vs 0.34). Across the myocardium the mean $B_{1}^{+}$ and beta were 1.05 and 0.69, respectively.

**FIGURE 8:**
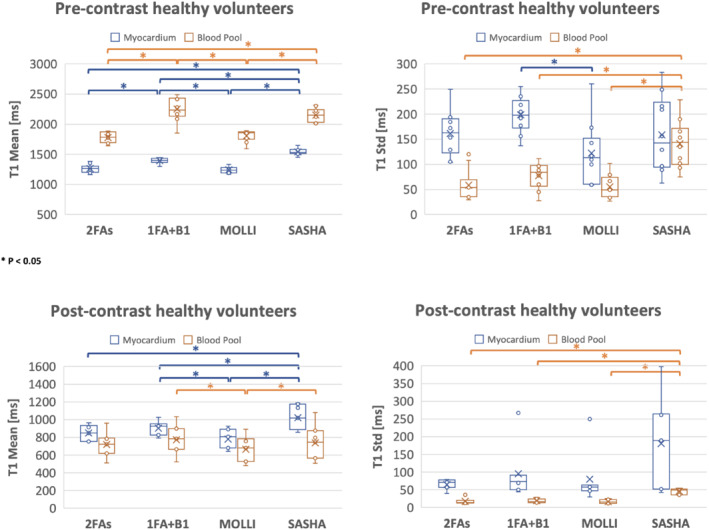
T1 quantifications for healthy volunteers. Four groups' box and whisker plots from left to right correspond to T1 values from 2FAs technique, 1FA + B1^+^ technique, MOLLI, and SASHA. Blue indicates the myocardium T1 values and orange represents the blood pool T1 values. For pre‐contrast *N* equals to 10, while for post‐contrast *N* equals to 7. * indicates *p* < 0.05. *x* represents mean value. Some data points with similar values appear overlapped due to resolution of the figure

Figure 8 compares the $T_{1}$ values for myocardium and blood pool among the 2FAs, 1FA + $B_{1}^{+}$, MOLLI, and SASHA techniques for healthy volunteers. For pre‐contrast results (*N* = 10), the mean myocardium $T_{1}$ values for 1FA + $B_{1}^{+}$ were more similar to those from SASHA $T_{1}$ maps, which are known to more closely match IR‐SE than those from MOLLI. MOLLI is known to underestimate $T_{1}$ values, especially in pre‐contrast studies. The SD, indicating the precision of $T_{1}$ measurements, are slightly higher for 2FAs and 1FA + $B_{1}^{+}$ techniques, but with no significant difference from SASHA. This might be due to several factors, such as residual motion during the free‐breathing acquisitions. In terms of blood pool $T_{1}$ values, although there are slight differences in the point estimates, no significant differences were identified between 2FAs and MOLLI, 1FA + $B_{1}^{+}$ and SASHA. Similar results were seen in the post‐contrast cases (*N* = 7). Furthermore, the precision of myocardium SASHA $T_{1}$ values were lower than the other three techniques. For the seven healthy subjects who underwent repeated measurements of native $T_{1}$, no significant difference was observed for 2FAs (*p* = 0.58, *p* = 0.56) and 1FA + $B_{1}^{+}$ (*p* = 0.39, *p* = 0.24) techniques in terms of both myocardium and blood pool $T_{1}$ values.

**FIGURE 9:**
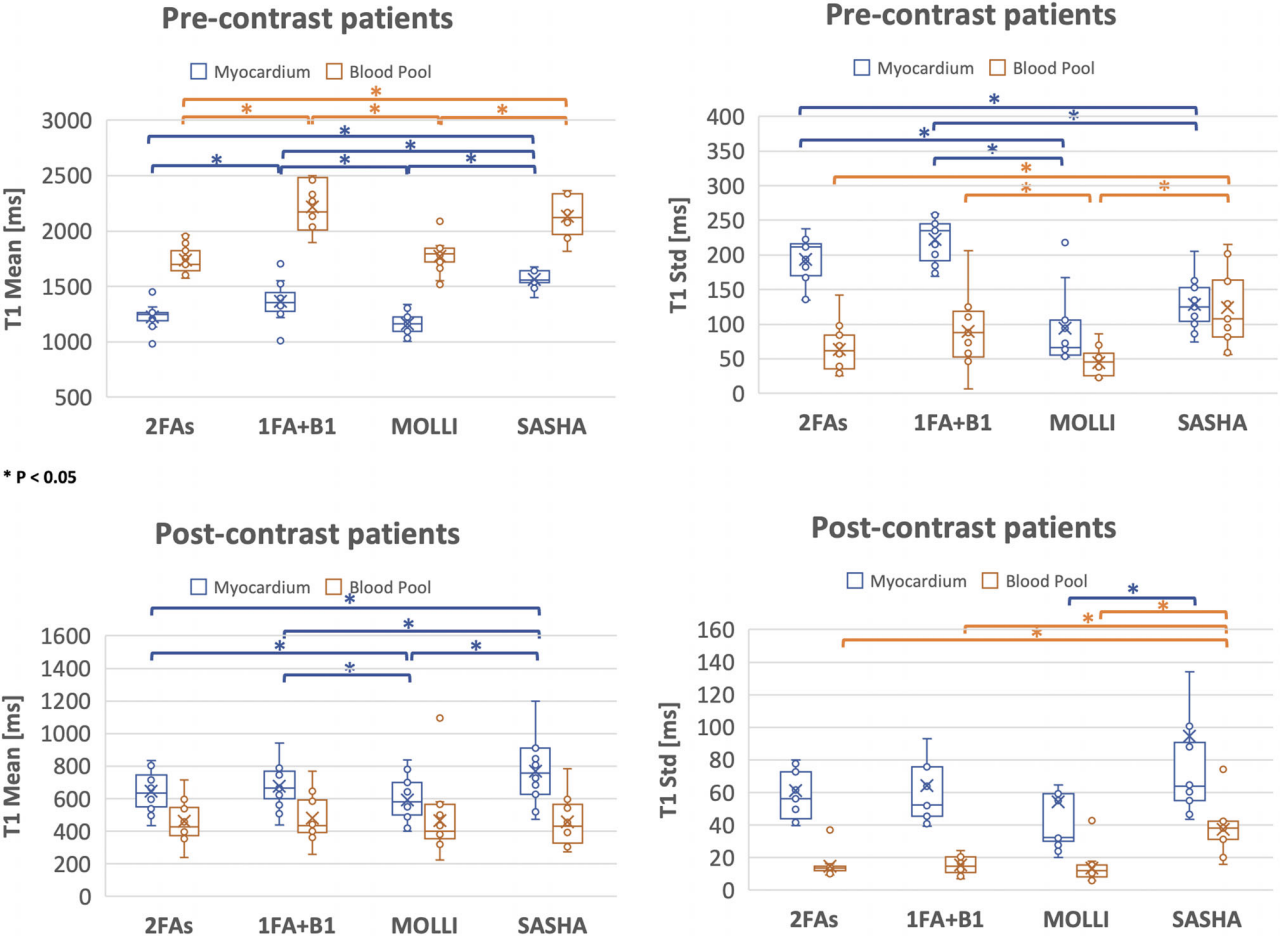
T1 quantifications for patients. Four groups' box and whisker plots from left to right correspond to T1 values from 2FAs technique, 1FA + B1^+^ technique, MOLLI, and SASHA. Blue indicates the myocardium T1 values and orange represents the blood pool T1 values. For both pre‐contrast and post‐contrast *N* equals to 15. * indicates *p* < 0.05. *x* represents mean value. Some data points with similar values are overlapping due to the resolution of the figure

Analogous comparisons are made for the patient group in Figure 9. For pre‐contrast results, both myocardium and blood pool $T_{1}$ mean and SD values showed similar trends compared to the ones from healthy volunteers, except that the individual data points are more variable. For post‐contrast, variability of individual points was also observed. One regional $T_{1}$ variation example from a cardiomyopathy infiltrative patient can be seen in Supporting Information Figure S6. Table 1 shows mean and SD of $T_{1}$ values in patient group. Furthermore, to compare the ECV for subjects who performed both pre‐ and post‐contrast acquisitions, global lambda (λ) among the mentioned four techniques were compared in Supporting Information Figure S7. Overall, the mean (±SD) image quality scores for 2FAs, 1FA + $B_{1}^{+}$, MOLLI, and SASHA are 3.35 (±0.61), 3.23 (±0.61), 4.14 (±0.74), and 3.96 (±0.60), where the breath‐hold ECG‐gated SSFP MOLLI and SASHA have better image quality compare to the free‐breathing self‐gated spoiled‐GRE proposed techniques (*p* < 0.05).

**TABLE 1:** Patient group T1 mean and SD comparison

Patients (*N* = 15)	2FA myocardium	1FA + B1 myocardium	MOLLI myocardium	SASHA myocardium	2FA blood pool	1FA + B1 blood pool	MOLLI blood pool	SASHA blood pool
Pre‐contrast T1 mean [ms]	1226.4	1364.9	1160.3	1564.1	1739.0	2219.1	1772.8	2132.9
Pre‐contrast T1 SD [ms]	193.7	222.8	94.6	128.5	64.0	90.0	44.4	123.8
Post‐contrast T1 mean [ms]	647.7	675.7	594.0	768.1	460.5	482.99	466.9	458.3
Post‐contrast T1 SD [ms]	61.3	64.2	54.3	94.4	14.7	15.3	13.6	37.2

## 4 DISCUSSION

We developed a strategy to acquire $B_{1}^{+}$ and $T_{1}$ maps in a free‐breathing, continuous inversion‐recovery spiral acquisition. This provides an alternative method to measure $T_{1}$ using a continuous Look‐Locker acquisition as compared to the dual excitation flip‐angle technique.^15^ For phantom results, both 1FA + $B_{1}^{+}$ and 2FAs techniques were more accurate than MOLLI when compared to the gold‐standard IR‐SE. For in‐vivo studies of both healthy volunteer and patient groups, myocardial $T_{1}$ values from 2FAs technique were closer to the clinically used MOLLI, whereas the results from 1FA + $B_{1}^{+}$ method were more similar to the standard breath‐held SASHA technique, demonstrating increased accuracy in measuring $T_{1}$. This difference could be due to the separate acquisition of more accurate $B_{1}^{+}$ maps in the 1FA + $B_{1}^{+}$ technique. Furthermore, in terms of pre‐ and post‐contrast acquisitions at the same slice position, the 1FA + $B_{1}^{+}$ technique only requires acquisition of a single $B_{1}^{+}$ map, which helps to further speed up the scan.

In this study, $B_{1}^{+}$ maps were typically acquired with 1.5 × 1.5 mm^2^ in‐plane spatial resolution. This provided us the flexibility to reconstruct the final $B_{1}^{+}$ maps over a range of spatial resolutions during technique development. Reconstructing the $B_{1}^{+}$ maps with 10 × 10 mm^2^ resolution offered adequate resolution given the low‐frequency variations in the $B_{1}^{+}$ field while improving SNR. After choosing the desired spatial resolution, we modified the spiral trajectories as shown in Figure 4F–J, to directly acquire $B_{1}^{+}$ maps with 10 × 10 mm^2^ resolution. By keeping the same readout duration for each spiral, the SNR of the $B_{1}^{+}$ maps were improved. An assumption of our $B_{1}^{+}$ mapping strategy is that the effects of respiratory motion are not significant given that the *B*_1_ field is slowly varying. A self‐gated and respiratory corrected approach for acquiring the field map could be developed to further mitigate any effects from respiratory motion.

However, there are several things that can be further improved in terms of the 1FA + $B_{1}^{+}$ technique. Firstly, since we only acquired the data to generate a low flip angle (3°) $T_{1}^{*}$ map, unlike the 2FAs strategy,^15^ where 15° data were acquired in order to reconstruct a second $T_{1}^{*}$ map, the 1FA + $B_{1}^{+}$ technique does not produce as high‐quality cine and LGE images as the 2FAs approach due to the low flip angle. While in this study our goal was to look at the accuracy of the 1FA + $B_{1}^{+}$ technique, there is no fundamental reason that the 2FAs approach could not be combined with the $B_{1}^{+}$ mapping proposed to also obtain higher SNR cine and LGE images. Higher flip angles will result in a shorter $T_{1}^{*}$. As the $T_{1}$‐weighted data points occur in diastole of subsequent heart beats that are separately by 600–1000 ms for typical heart rates, a shorter $T_{1}^{*}$ will result in less data points on the steep portion of the $T_{1}^{*}$ recovery curve, which is most sensitive to the differences in $T_{1}$. This could affect both the accuracy and precision of the $T_{1}$ measurements. In this study, the 3° flip angle was chosen to match that of the 2FAs strategy, and could be further optimized to trade off $T_{1}$ accuracy for cine and LGE image quality. We have previously demonstrated that good quality cine images can be acquired with similar continuous golden‐angle spiral approach with flip angle of 7 degrees.^21^ Furthermore, the $B_{1}^{+}$ mapping approach could be combined with a multiple‐flip angle strategy. This requires further study on the choice of flip angles and the timing to perform $B_{1}^{+}$ mapping. The image quality of the proposed $T_{1}$ mapping technique can sometimes be limited by residual motion artifacts and lower SNR. Residual motion artifacts could be lessened by using advanced non‐rigid image reconstruction. However, for 2D imaging, the effect of through‐plane motion and blood flow on calculated $T_{1}$ values cannot be corrected, due to the complex motion of the heart and blood flow in the LV cavity. Therefore, it remains a challenge for accurate $T_{1}$ quantification using continuous 2D acquisition techniques. 3D imaging may reduce the effect of through‐plane motion on $T_{1}$ accuracy, but this requires further study. In terms of B0 inhomogeneity, B0 correction algorithms,^22^, ^23^, ^24^, ^25^ shorter spiral interleaves, or 3D acquisition with thinner partitions that allows for B0‐correction in the through‐plane direction may reduce off‐resonance artifacts. Improved techniques for motion guided reconstruction could improve image quality. Also, the acquisitions are spoiled‐GRE, which have a lower inherent SNR but are less sensitive to off‐resonance, as compared to SSFP in MOLLI and SASHA.

Moreover, although there are some biases in the blood pool $T_{1}$ values from the proposed techniques compared to MOLLI and SASHA, the differences are not significant. As shown in Supporting Information Figure S7, the proposed techniques have comparable lambda (proportional to ECV) to that from MOLLI. We observed some bias in the 2FAs β map comparing to directly measured BS $B_{1}^{+}$ map in the in‐vivo case. This might be due to inflow of blood into the slice, and effects of through‐plane motion of the slice resulting from respiration. For the cavity, inflow of blood results in a different magnetization history. Similarly, through‐plane motion will result in a different magnetization history both due to bulk motion and movement of the anatomy across the slice profile. This disruption in the magnetization results in bias of the 2FAs β map and $T_{1}$ map. For the BS $B_{1}^{+}$ mapping technique, the positive and negative Fermi pulses are acquired in an interleaved manner to improve robustness to motion. Second, the BS pulses are applied non‐selectively, and the $B_{1}^{+}$ field is slowly varying, so this technique might be less sensitive to motion and flow artifacts, but may still have sensitivity to through‐plane motion as they are acquired in free‐breathing over 2 s, which might affect the accuracy of these techniques in vivo. The main discrepancy between the 2FAs and BS $B_{1}^{+}$ techniques is related to the difference in $B_{1}^{+}$ scale factors derived by these two methods. The inherent sensitivity of the $T_{1}$ values to $B_{1}^{+}$ scale factor variations remain a challenge for any continuous parametric mapping technique that relies on quantifying $T_{1}^{*}$, since it is inherent function of both $B_{1}^{+}$ and $T_{1}$. We acknowledge that given $T_{1}$ and $B_{1}^{+}$ are in the mono‐exponential recovery term $T_{1}^{*}$ for continuously acquired parametric mapping techniques, and as M0 is not sampled during continuous acquisition, the $B_{1}^{+}$ sensitivity of this, like other similar techniques, is a significant limitation. While the BS $B_{1}^{+}$ mapping provides more accurate $T_{1}$ values, the exact $B_{1}^{+}$ map accuracy cannot be validated in vivo due to lack of a suitable reference $B_{1}^{+}$ mapping technique. Given our current data and the lack of an accepted $B_{1}^{+}$ reference standard, it is difficult to quantify the $B_{1}^{+}$ accuracy in vivo. Last, the 1FA + $B_{1}^{+}$ technique requires a motion‐correction strategy between $B_{1}^{+}$ and $T_{1}^{*}$ maps. This is less reliable than the 2FAs strategy, where image registration is performed only on $T_{1}^{*}$ maps.

## 5 CONCLUSIONS

In a single acquisition, a free‐breathing BS shift $B_{1}^{+}$ map, and a self‐gated $B_{1}^{+}$ map and slice profile corrected *T*_1_ map were acquired. This technique was compared to the prior dual‐flip angle approach and yielded more accurate *T*_1_ values in the myocardium.

## Supporting information


**Figure S1** Dictionary learning reconstruction. Images in the first row show the images after direct gridding. Images in the second row show the dictionary learning reconstructed results. These dictionary learning reconstruction images were used to fit to the 3‐parameter model to obtain the T1* map.
**Figure S2** Look‐up table as a function of B1, T1* and T1 at 3 degrees flip angle. (a) shows the relationship between T1 and T1* at certain B1 values. (b) shows the relationship between B1 and T1* at certain T1 values.
**Figure S3** Bland‐Altman plots of phantom T1 values
**Figure S4** Phantom T1 value comparison between with and without considering slice profile effect. (a)(b) show the 2FAs T1 map without and with considering slice profile, while (c)(d) represent the 1FA+B1+ T1 map without and with considering slice profile. (e) indicate the IR‐SE T1 map. (f) showed the T1 values comparison from 9 tubes among the four techniques.
**Figure S5** B1 comparisons from 1FA+B1 B1 map and 2FAs beta map at the myocardium and blood pool across all the subjects during pre‐contrast.
**Figure S6** A cardiomyopathy infiltrative patient example at post‐contrast demonstrating regional T1 variation at basal lateral.
**Figure S7** Global lambda comparison of healthy volunteers' and patients' group. For healthy volunteers' group, SASHA lambda has a significant difference compared to all other techniques (*p* < 0.05). For patients' group, SASHA lambda has a significant difference compared to MOLLI (*p* < 0.05).

## References

[1] Liu S , Han J , Nacif MS , et al. Diffuse myocardial fibrosis evaluation using cardiac magnetic resonance T1 mapping: sample size considerations for clinical trials. J Cardiovasc Magn Reson. 2012;14:90. 23272704 10.1186/1532-429X-14-90 PMC3552738

[2] Araujo‐Filho JAB , Assuncao AN Jr , Tavares de Melo MD , et al. Myocardial T1 mapping and extracellular volume quantification in patients with left ventricular non‐compaction cardiomyopathy. Eur. Hear. J. Cardiovasc. Imaging. 2018;19:888‐895. 10.1093/ehjci/jey022 29518212

[3] Robinson AA , Chow K , Salerno M . Myocardial T1 and ECV measurement: underlying concepts and technical considerations. JACC Cardiovasc Imaging. 2019;12:2332‐2344. 31542529 10.1016/j.jcmg.2019.06.031 PMC7008718

[4] Messroghli DR , Radjenovic A , Kozerke S , Higgins DM , Sivananthan MU , Ridgway JP . Modified look‐locker inversion recovery (MOLLI) for high‐resolution T 1 mapping of the heart. Magn Reson Med. 2004;52:141‐146. 15236377 10.1002/mrm.20110

[5] Chow K , Flewitt JA , Green JD , Pagano JJ , Friedrich MG , Thompson RB . Saturation recovery single‐shot acquisition (SASHA) for myocardial T1 mapping. Magn Reson Med. 2014;71:2082‐2095. 23881866 10.1002/mrm.24878

[6] Shaw JL , Yang Q , Zhou Z , et al. Free‐breathing, non‐ECG, continuous myocardial T _1_ mapping with cardiovascular magnetic resonance multitasking. Magn Reson Med. 2018;81:2450‐2463. 30450749 10.1002/mrm.27574 PMC6372325

[7] Qi H , Jaubert O , Bustin A , et al. Free‐running 3D whole heart myocardial T _1_ mapping with isotropic spatial resolution. Magn Reson Med. 2019;82:1331‐1342. 31099442 10.1002/mrm.27811 PMC6851769

[8] Chow K , Yang Y , Shaw P , Kramer CM , Salerno M . Robust free‐breathing SASHA T1 mapping with high‐contrast image registration. J Cardiovasc Magn Reson. 2016;18:1‐14. 27535744 10.1186/s12968-016-0267-9 PMC4989502

[9] Yarnykh VL . Optimal radiofrequency and gradient spoiling for improved accuracy of T1 and B1 measurements using fast steady‐state techniques. Magn Reson Med. 2010;63:1610‐1626. 20512865 10.1002/mrm.22394

[10] Stikov N , Boudreau M , Levesque IR , Tardif CL , Barral JK , Pike GB . On the accuracy of T1 mapping: searching for common ground. Magn Reson Med. 2015;73:514‐522. 24578189 10.1002/mrm.25135

[11] Zhou R , Weller DS , Yang Y , Wang J , Mugler JP , Salerno M . Free‐breathing continuous cine and T1 mapping acquisition using a motion‐corrected dual flip angle inversion‐recovery spiral technique at 3T.

[12] Sacolick LI , Wiesinger F , Hancu I , Vogel MW . B1 mapping by Bloch‐Siegert shift. Magn Reson Med. 2010;63:1315‐1322. 20432302 10.1002/mrm.22357 PMC2933656

[13] Hamilton JI , Jiang Y , Chen Y , et al. MR fingerprinting for rapid quantification of myocardial T1, T2, and proton spin density. Magn Reson Imaging. 2017;1458:1446‐1458. 10.1002/mrm.26216 PMC5045735 27038043

[14] Heidenreich JF , Weng AM , Donhauser J , et al. T1‐ and ECV‐mapping in clinical routine at 3 T: differences between MOLLI. ShMOLLI and SASHA BMC Med Imaging. 2019;19:1‐9. 10.1186/s12880-019-0362-0 PMC6676542 31370821

[15] Zhou R , Weller DS , Yang Y , et al. Dual‐excitation flip‐angle simultaneous cine and T1 mapping using spiral acquisition with respiratory and cardiac self‐gating. Magn Reson Med. 2021;86:82‐96. 33590591 10.1002/mrm.28675 PMC8849625

[16] Fessler JA . On NUFFT‐based gridding for non‐Cartesian MRI. J Magn Reson. 2007;188:191‐195. 17689121 10.1016/j.jmr.2007.06.012 PMC2121589

[17] Walsh DO , Gmitro AF , Marcellin MW . Adaptive reconstruction of phased array MR imagery. Magn Reson Med. 2000;43:682‐690. 10800033 10.1002/(sici)1522-2594(200005)43:5<682::aid-mrm10>3.0.co;2-g

[18] Lesch A , Schlöegl M , Holler M , Bredies K , Stollberger R . Ultrafast 3D Bloch–Siegert B1^+^‐mapping using variational modeling. Magn Reson Med. 2019;81:881‐892. 30444294 10.1002/mrm.27434 PMC6491998

[19] Captur G , Gatehouse P , Kellman P , et al. A T1 and ECV phantom for global T1 mapping quality assurance: the T1 mapping and ECV standardisation in CMR (T1MES) program. J Cardiovasc Magn Reson. 2016;18:1‐4. 27660042 10.1186/s12968-016-0280-z PMC5034411

[20] Yang Y , Kramer CM , Shaw PW , Meyer CH , Salerno M . First‐pass myocardial perfusion imaging with whole‐heart coverage using L1‐SPIRiT accelerated variable density spiral trajectories. Magn Reson Med. 2016;76:1375‐1387. 26538511 10.1002/mrm.26014 PMC4860174

[21] Zhou R , Weller DS , Yang Y , Wang J , Mugler J , Salerno M . Single acquisition of cine images and T1 maps using a free‐breathing respiratory motion‐corrected spiral technique at 3T. Proceedings of the 23rd Annual SCMR Scientific Sessions. 2020:1592‐1595.

[22] Man L , Pauly JM , Macovski A . Improved automatic off‐resonance correction without a field map in spiral imaging. Magn Reson Med. 1997;37:906‐913. 9178243 10.1002/mrm.1910370616

[23] Noll DC , Pauly JM , Meyer CH , Nishimura DG , Macovskj A . Deblurring for non‐2D fourier transform magnetic resonance imaging. Magn Reson Med. 1992;25:319‐333. 1614315 10.1002/mrm.1910250210

[24] Chen W , Meyer CH . Fast automatic linear off‐resonance correction method for spiral imaging. Magn Reson Med. 2006;56:457‐462. 16810696 10.1002/mrm.20973

[25] Smith TB , Nayak KS . Automatic off‐resonance correction in spiral imaging with piecewise linear autofocus. Magn Reson Med. 2013;69:82‐90. 22457262 10.1002/mrm.24230

