# Supplementary material for: Free‐breathing self‐gated continuous‐IR spiral T1 mapping: Comparison of dual flip‐angle and Bloch‐Siegert B1‐corrected techniques
Source: Magn Reson Med. 2022 Apr 28;88(3):1068–80. doi: 10.1002/mrm.29269 (PMC9325422; doi:10.1002/mrm.29269)
Supplement: Supplementary file 1 — Figure S1 Dictionary learning reconstruction. Images in the first row show the images after direct gridding. Images in the second row show the dictionary learning reconstructed results. These dictionary learning reconstruction images were used to fit to the 3‐parameter model to obtain the T1* map. Figure S2 Look‐up table as a function of B1, T1* and T1 at 3 degrees flip angle. (a) shows the relationship between T1 and T1* at certain B1 values. (b) shows the relationship between B1 and T1* at certain T1 values. Figure S3 Bland‐Altman plots of phantom T1 values Figure S4 Phantom T1 value comparison between with and without considering slice profile effect. (a)(b) show the 2FAs T1 map without and with considering slice profile, while (c)(d) represent the 1FA+B1+ T1 map without and with considering slice profile. (e) indicate the IR‐SE T1 map. (f) showed the T1 values comparison from 9 tubes among the four techniques. Figure S5 B1 comparisons from 1FA+B1 B1 map and 2FAs beta map at the myocardium and blood pool across all the subjects during pre‐contrast. Figure S6 A cardiomyopathy infiltrative patient example at post‐contrast demonstrating regional T1 variation at basal lateral. Figure S7 Global lambda comparison of healthy volunteers' and patients' group. For healthy volunteers' group, SASHA lambda has a significant difference compared to all other techniques (p < 0.05). For patients' group, SASHA lambda has a significant difference compared to MOLLI (p < 0.05). [file MRM-88-1068-s001.docx]

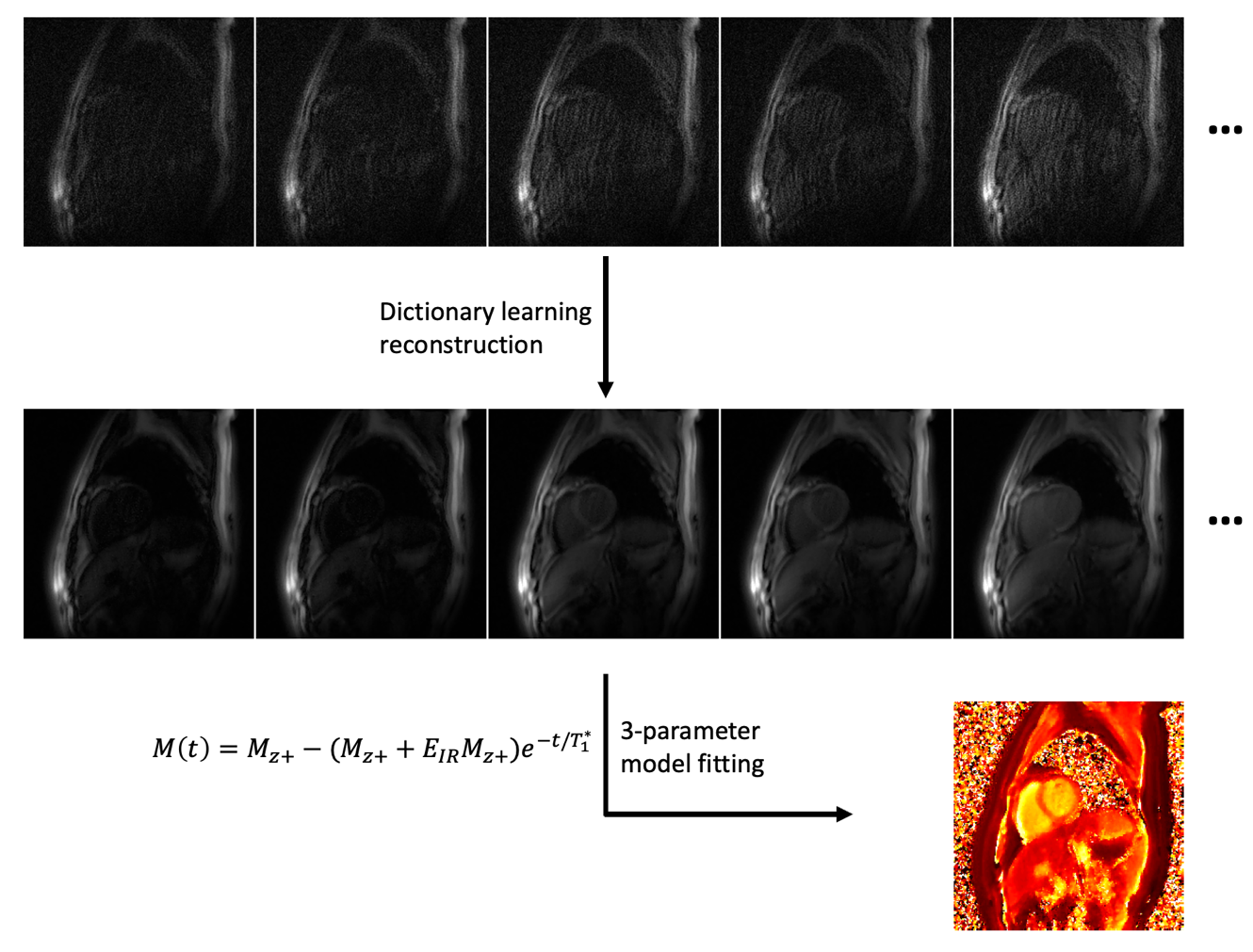


Supporting Information Figure S1: Dictionary learning reconstruction. Images in the first row show the images after direct gridding. Images in the second row show the dictionary learning reconstructed results. These dictionary learning reconstruction images were used to fit to the 3-parameter model to obtain the T1* map.


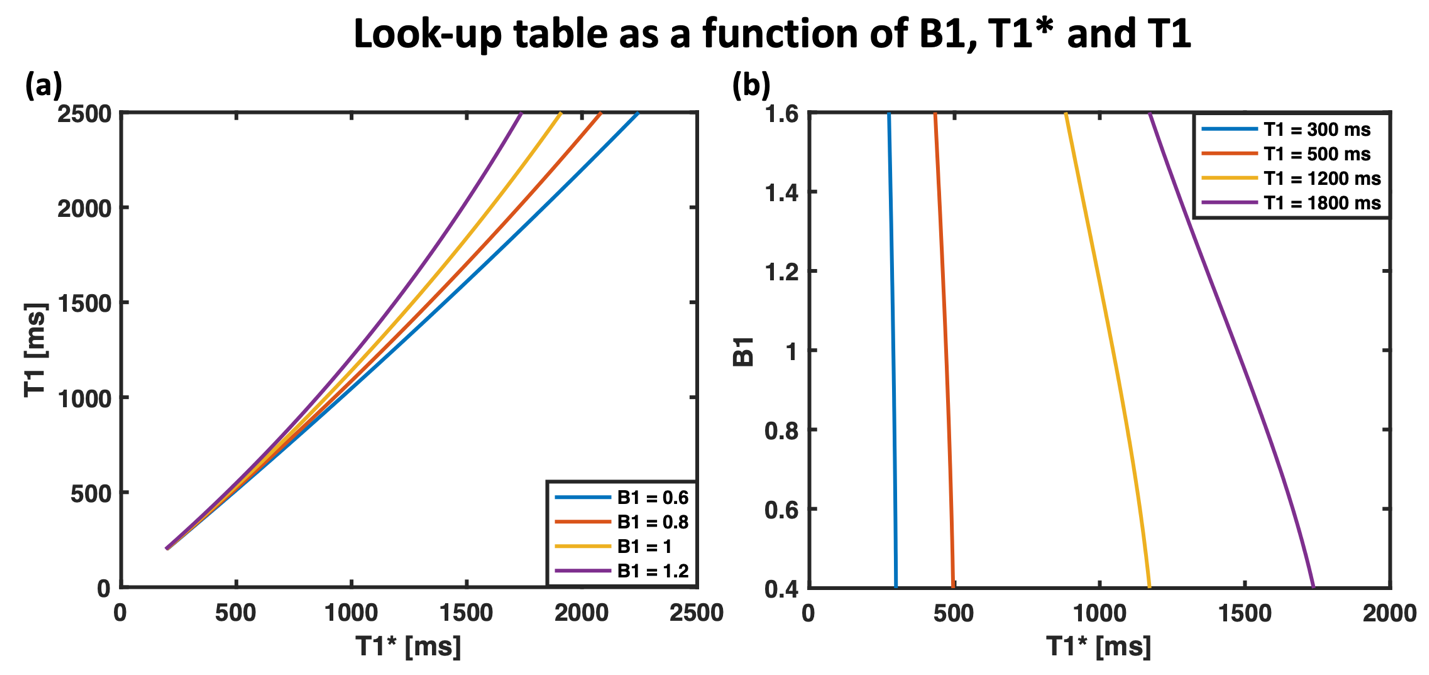


Supporting Information Figure S2: Look-up table as a function of B1, T1* and T1 at 3 degrees flip angle. (a) shows the relationship between T1 and T1* at certain B1 values. (b) shows the relationship between B1 and T1* at certain T1 values.

*
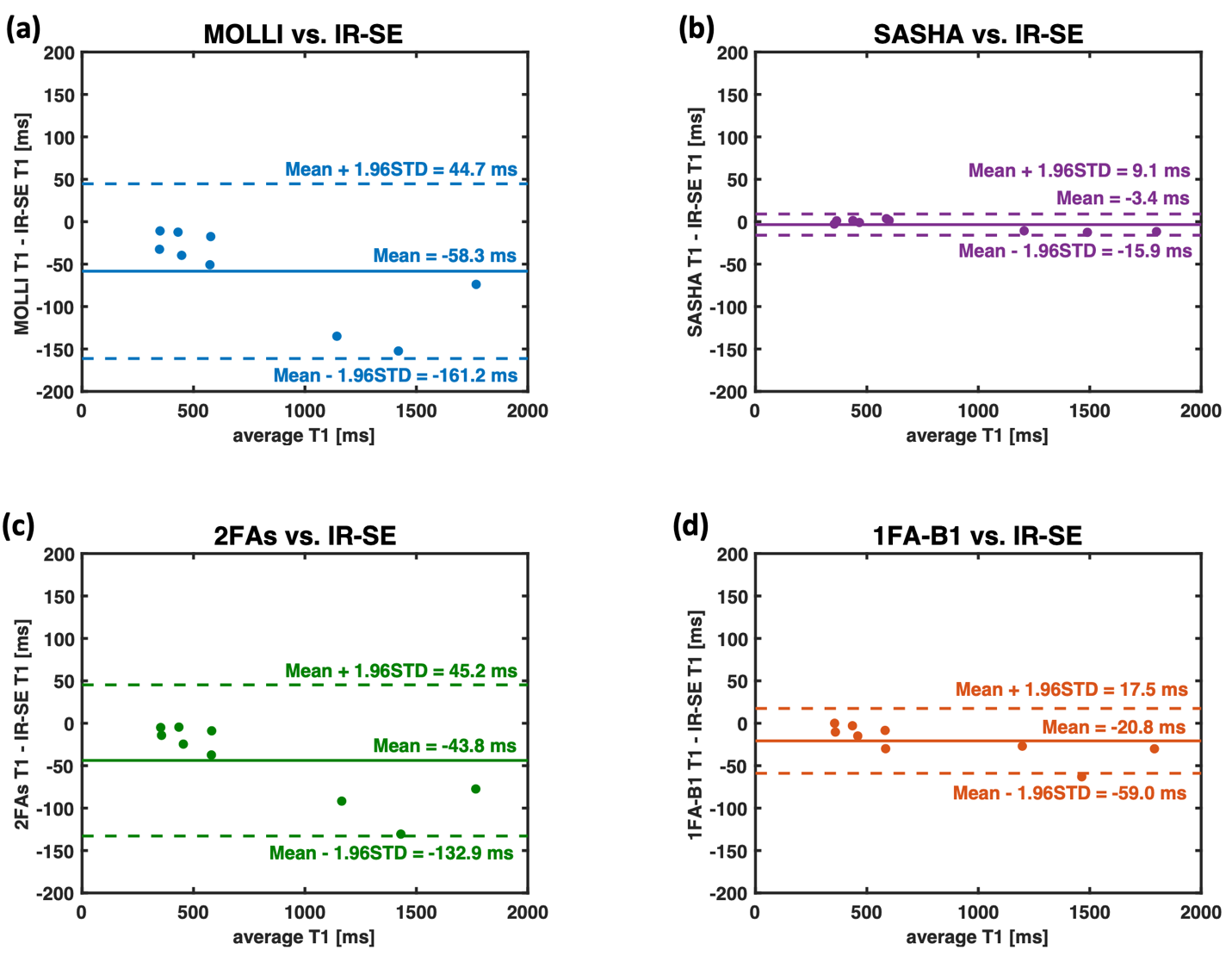
*

Supporting Information Figure S3: Bland-Altman plots of phantom T1 values.


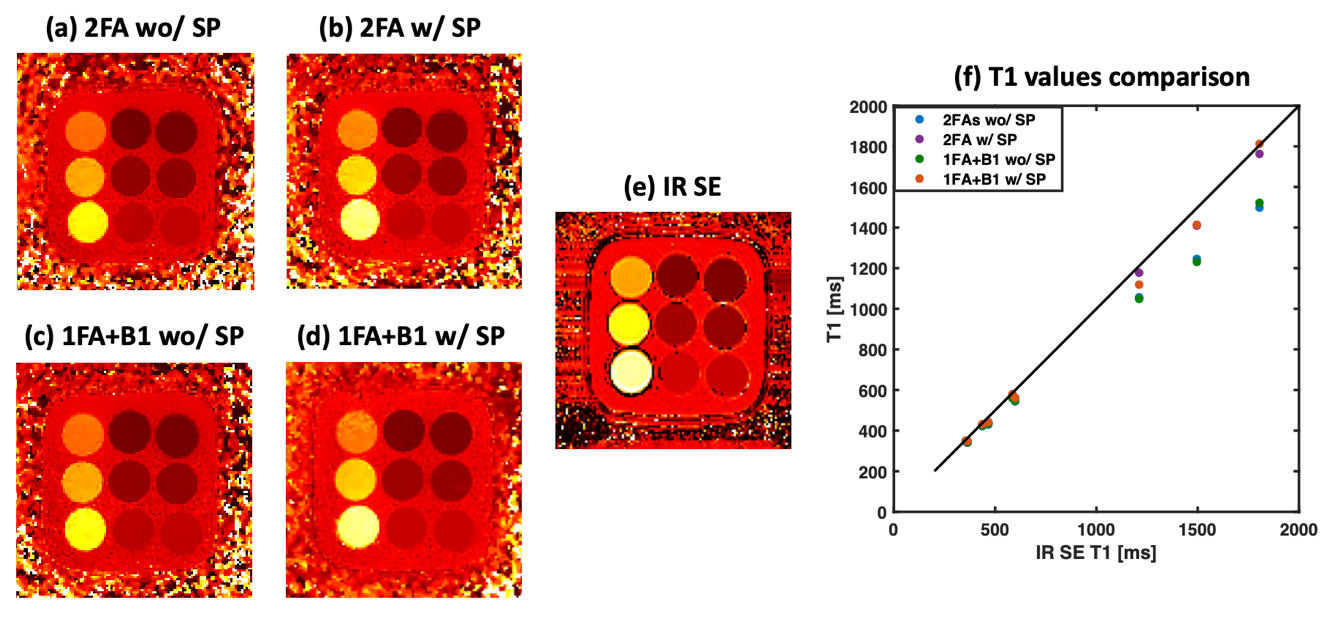


Supporting Information Figure S4: Phantom T1 value comparison between with and without considering slice profile effect. (a)(b) show the 2FAs T1 map without and with considering slice profile, while (c)(d) represent the 1FA+B1+ T1 map without and with considering slice profile. (e) indicate the IR-SE T1 map. (f) showed the T1 values comparison from 9 tubes among the four techniques.


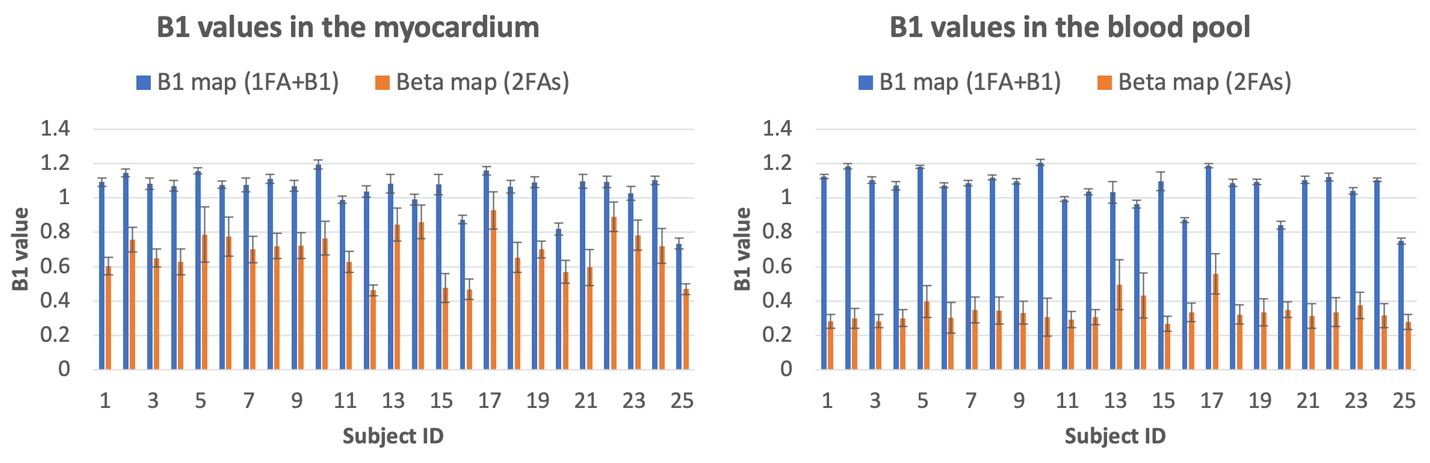


Supporting Information Figure S5: B1 comparisons from 1FA+B1 B1 map and 2FAs beta map at the myocardium and blood pool across all the subjects during pre-contrast.


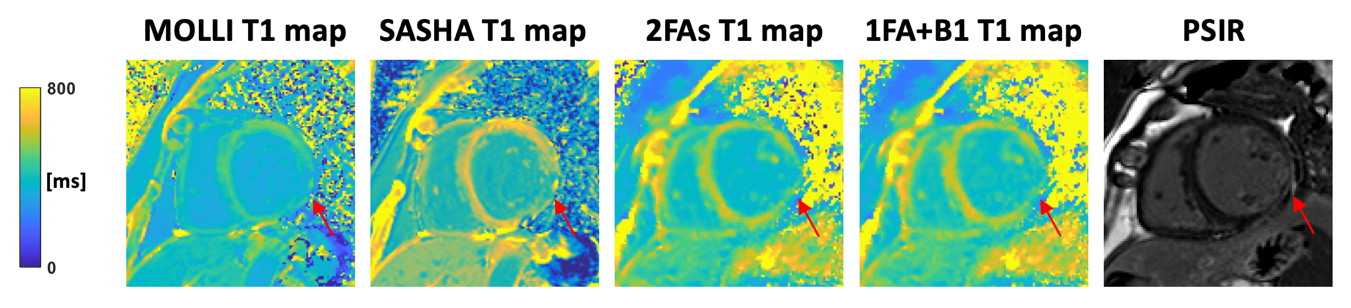


Supporting Information Figure S6: A cardiomyopathy infiltrative patient example at post-contrast demonstrating regional T1 variation at basal lateral.

**
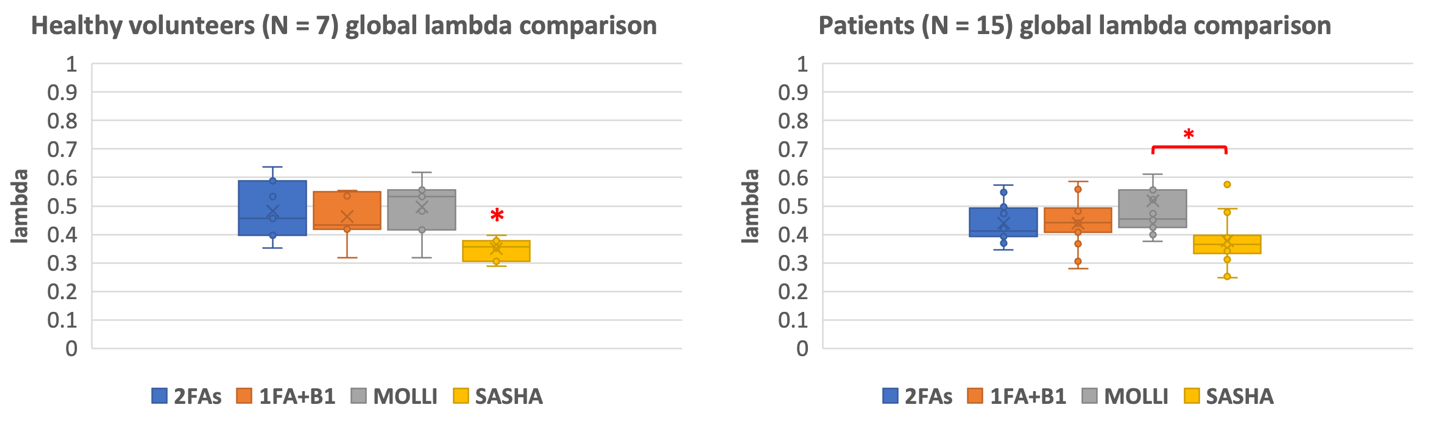
**

Supporting Information Figure S7: Global lambda comparison of healthy volunteers’ and patients’ group. For healthy volunteers’ group, SASHA lambda has a significant difference compared to all other techniques (p < 0.05). For patients’ group, SASHA lambda has a significant difference compared to MOLLI (p < 0.05).
